# Effects of plant density on alfalfa (*Medicago sativa* L.) seed yield in western Heilongjiang areas

**DOI:** 10.1515/biol-2022-0792

**Published:** 2023-12-16

**Authors:** Xiaolong Wang, Peng Zhong, Zhao Yang, Yongcai Lai, Shasha Li, Hua Chai, Yanxia Xu, Yue Wu, Jianli Wang

**Affiliations:** Branch of Animal Husbandry and Veterinary of Heilongjiang Academy of Agricultural Sciences, Qiqihar-161005, China; Postdoctoral Research Workstation of Heilongjiang Academy of Agricultural Sciences, Harbin-150086, China; Institute of Grass Science of Heilongjiang Academy of Agricultural Sciences, Harbin-150086, China

**Keywords:** alfalfa, row spacing, plant spacing, yield components, seed yield

## Abstract

Alfalfa (*Medicago sativa* L.) is known as the “king of forages”. The aim of the current study is to determine the optimum planting density as the key cultivation technique for high yield of alfalfa seed. Alfalfa variety (Longmu 801) was planted in experimental fields from 2014 to 2017. In the planting density test, the row spacing was 65, 80, and 95 cm, and the plant spacing was 30, 45, 60, 75, and 90 cm. The seed yield and yield components in the row spacing and plant spacing tests were measured. On the basis of 3 years average of the experimental data, the highest seed yield of 225.49 kg ha^−1^ was obtained with row spacing vs plant spacing of 65 and 60 cm, respectively. Correlation analysis showed a significant positive correlation between the racemes per stem, pods per raceme, pods per stem, seeds per pod, and the seed yield. These results suggested that Longmu 801 should be cultivated with 65 cm row spacing and 60 cm plant spacing to maximize seed yields in western Heilongjiang areas.

## 1 Introduction

Alfalfa (*Medicago sativa* L.) is one of the most important legume forage that is grown worldwide [1,2]. Due to high yield and rich nutritional value, alfalfa is known as the “king of forages” [3,4,5]. As the alfalfa planting area has been expanding continuously, the demand for high-quality alfalfa seed has also been increasing gradually. To this flow, the supply of alfalfa seeds however has become a key limiting factor towards the development of alfalfa [6,7]. Therefore, there is an urgent need to solve the problem of generally low seed yields in alfalfa seed production. Many factors including climatic conditions, soil fertility, agronomic measures, and genetic factors affect the increase in alfalfa seed yields [8,9,10]. Among, suitable agronomic measures including cultivation methods and planting density are important factors in increasing seed yield [11,12,13]. Therefore, the most suitable planting density is the key cultivation technology for the high yield of alfalfa seeds.

In the region of northeast China, the seed yield is low in recent years, which limits seed production potential of alfalfa. Seed yield is a very complex trait which depends upon a number of factors. Among those, the appropriate planting density can promote stem growth and increase the reproductive coefficient, thereby increasing the yield of alfalfa seeds [14]. Because in alfalfa, the planting density has a strong effect on the production of number of branches, inflorescences, and pods, and seed yield. Compared with other un-thinned plants of alfalfa, thinned plants were found more easily pollinated by insect. Some researchers have recommended that row spacing varied from 20 to 91 cm, and plant density could affect significantly on seed yield components, and it may be a better cultivation technique for achieving high seed yields [15,16,17]. But only few research works had considered the effect of plant spacing on alfalfa seed yield [17,18]. The plant density of alfalfa significantly increased the quantity of stems per square meter (m^2^), pods per stem, racemes per stem, and pods per raceme, but not significant on alfalfa seed weight [15]. Besides, row spacing and plant spacing can influence seed yield for several years. At present, the alfalfa planting area is increasing rapidly and its seed production technology is backward, it is of important ecological and economic significance to explore the suitable planting density (row spacing and plant spacing) of alfalfa seed production in western Heilongjiang areas. Therefore, the current study was aimed to explore the effect of planting density on alfalfa seed yield, and then determine the optimal planting density for alfalfa seed high-yield cultivation in Heilongjiang, China and other similar upper-latitude semiarid environments.

## 2 Materials and methods

The field experiment was conducted at the Heilongjiang Academy of Agricultural Sciences, Qiqihar (47°15′ N, 123°41′ E), Heilongjiang, China from 2014 to 2017. The soil basic nutrient index (0–30 cm depth), including organic matter (19.89 g kg^−1^), total N (1.19 g kg^−1^), available P (10.60 mg kg^−1^), available K (124.84 mg kg^−1^), and pH value of 7.40 (1:2.5 soil:water) was determined. The average annual rainfall is approximately 352 mm. The average temperature is 4.0°C. The previous crop was maize (*Zea mays* L.) before cultivating the alfalfa field experiment.

The field experiment was conducted following a complete randomized block design. The experimental plot was set up for 3 replicates. Each replication had 15 treatment combinations. The field experiment treatments were arranged as 3 × 5 factorial combinations of three row spacings (R) (65, 80, and 95 cm), five plant spacings (P) (30, 45, 60, 75, and 90 cm). The size of each experimental plot was 3 × 5 m (15 m^2^), having 1 m spacing distance between the adjacent plots. The sowing seeds of Longmu 801 variety was provided by Heilongjiang Academy of Agricultural Sciences, China. The adaptability of Longmu 801 alfalfa was very high with wide application of its cultivation in the western Heilongjiang areas. The field experiment was started in 2014. Alfalfa seed yield was tested in experimental fields from 2015 to 2017. The sowing depth of alfalfa seeds was 1–2 cm, with 5–6 seeds per hole. Fertilizer was not applied during the establishment of the experimental field. The test plot was sown on 10 Jun, 2014 and the irrigation was carried out four times on 25 Jun, 10 July, 25 July, and 25 November every year, respectively. Each clump in the test plot was thinned to two plants per clump during the re-green stage in 2015. The test plots were hand weeded during the alfalfa growing period.

Alfalfa seed yield was determined by hand harvesting the seeds in three replicate samplings from each plot when 70% of the pods turned blackish brown. The flowering and harvesting dates of each year are shown in Table 1. Flowering dates were recorded when more than 50% of the stems had racemes. The seed samples collected from each plot was dried, threshed, sieved, and weighed and then put into mesh bags before laboratory testing. Alfalfa seed yield was calculated based on the seeds having 12% standard moisture content. The seed weight of each test plot (15 m^2^) was measured (repeated three times), and the average seed weight of the test plot was calculated to convert the seed weight per ha (kg ha^−1^). The yield components of alfalfa seeds, such as stems per m^2^, pods per stem, racemes per stem, pods per raceme, seeds per pod, and 1,000 seeds weight (g) were measured. Before alfalfa seed harvest, ten random clumps were sampled from each plot to measure the number of stems per clump. According to the average number of stems per clump, clump density was calculated and expressed as number of stems per m^2^. Thirty stems, 30 racemes, and 30 pods were randomly sampled from test plot to determine the number of racemes per stem, pods per stem, pods per raceme, and seeds per pod. Four random samples of seeds were dried at 80°C to constant moisture content, and then the 1,000 seeds weight was measured.

**Table 1:** Date of flowering and harvesting in 2015, 2016, and 2017

	Year		
	2015	2016	2017
Flowering date	15 June	17 June	18 June
Harvesting date	4 Aug	7 Aug	11 Aug

The test was conducted for 3 years (2015, 2016, and 2017) in Qiqihar, Heilongjiang, China. Years were considered a fixed effect because this experiment was aimed to explore whether test would increase yield of alfalfa seeds. The interaction of different years and two treatments (row spacing and plant spacing) were analyzed using Two-factors ANOVA (Table 2). Analysis of variance (ANOVA, *P* < 0.05) and correlation analysis (*P* < 0.05, *P* < 0.01) were performed using SAS 9.0 software (SAS Institute Inc, 2002, NC USA). The figure was drawn with Sigma Plot 12.5 software (Systat Software Inc, 2003, San Jose, CA USA).

**Table 2:** Years, plant spacing, and row spacing interactions on seed yield and yield components (stems per m^2^, racemes per stem, pods per stem, pods per raceme, seeds per pod, and 1,000 seeds weight) at Heilongjiang, China

Year	Source	df	Stems per m^2^	Racemes per stem	Pods per stem	Pods per raceme	Seeds per pod	1,000 seeds weight (g)	Seed yield (kg ha^−1^)
2015	Row (R)	2	**	*	NS	**	**	NS	**
	Plant (P)	4	**	**	**	**	**	NS	**
	R × P	8	*	**	**	*	*	NS	**
2016	Row (R)	2	**	NS	NS	NS	NS	NS	**
	Plant (P)	4	**	**	**	NS	*	*	**
	R × P	8	**	NS	NS	NS	**	NS	**
2017	Row (R)	2	**	NS	NS	*	**	NS	**
	Plant (P)	4	**	**	**	*	NS	NS	**
	R × P	8	**	NS	*	NS	NS	NS	**
2015–2017	Year (Y)	2	**	**	**	**	**	**	**
	Row (R)	2	**	NS	NS	**	**	NS	**
	Plant (P)	4	**	**	**	**	**	**	**
	Y × R	4	**	*	NS	NS	*	NS	**
	Y × P	8	**	**	**	**	NS	NS	**
	R × P	8	**	**	*	*	**	NS	**
	Y × R × P	16	*	NS	NS	**	**	NS	**

*0.05 level significant; **0.01 level significant; NS, not significant.

## 3 Results and discussion

### 3.1 Stems per m^2^

The quantity of stems per m^2^ of the plant spacing treatments over 3 years ranged from 81.7 to 189.8 (Table 3). However, a 30 cm plant spacing showed the highest number of stem production per m^2^. The greatest seed yields were maintained in the first harvest years despite having lesser stems per m^2^. Meanwhile, these findings were in agreement with Zhang et al. [17]. This suggested that the number of stems per m^2^ were not the only deciding factor in measuring seed yields, but may also be the result of racemes produced in thinner stands [19]. In the next 2 years, the highest seed yields were harvested from the treatment of 65 cm row spacing × 60 cm plant spacing. This result indicated that with the thinner plant density (row or plant spacing), the increased numbers of racemes per stem and pods per stem could not compensate for the decrease in the number of stems per m^2^.

**Table 3:** Average values for stems per m^2^, racemes per stem, and pods per stem in 2015, 2016, and 2017

Plant spacing (cm)	Row spacing (cm)	Stems per m^2^				Racemes per stem				Pods per stem			
		2015	2016	2017	Mean value	2015	2016	2017	Mean value	2015	2016	2017	Mean value
30	65**†**	174.5	201.7	278.0	218.1	16.8	24.6	20.2	20.5	49.61	41.9	42.3	44.6
	80	154.9	198.9	241.2	198.3	19.9	27.6	23.3	23.6	49.14	42.3	43.5	45.0
	95	120.4	141.5	197.6	153.2	22.9	30.8	24.6	26.1	54.33	45.8	44.4	48.2
45	65	137.6	165.9	250.7	184.7	18.3	29.2	23.9	23.8	48.97	49.2	45.1	47.8
	80	108.1	131.6	208.9	149.5	21.6	33.6	25.2	26.8	55.18	51.3	44.5	50.3
	95	82.5	120.9	149.7	117.7	22.0	33.9	26.1	27.3	56.54	54.3	47.4	52.7
60	65	110.8	164.4	215.2	163.5	30.4	37.3	25.7	31.1	66.71	63.3	50.4	60.1
	80	97.7	127.0	165.0	129.9	23.0	31.1	24.8	26.3	59.33	54.9	50.8	55.0
	95	69.9	98.7	120.3	96.3	23.3	31.6	25.1	26.7	50.56	58.5	49.6	52.9
75	65	87.7	112.0	163.6	121.1	24.3	31.9	27.6	27.9	59.09	56.1	48.4	54.5
	80	68.7	85.8	113.0	89.2	24.7	33.4	26.6	28.2	62.89	60.2	47.5	56.9
	95	64.7	74.0	96.9	78.5	30.4	34.8	25.9	30.4	64.96	65.2	47.2	59.1
90	65	73.1	91.2	115.5	93.3	30.5	34.9	28.1	31.2	66.44	63.1	54.4	61.3
	80	68.7	76.2	89.6	78.2	32.2	35.3	30.7	32.7	69.77	67.4	52.1	63.1
	95	61.7	74.5	84.6	73.6	35.0	42.1	31.9	36.3	74.72	70.6	52.4	65.9
**Row spacing treatments (mean value‡)**													
	65	116.7a	147.0a	204.6a	156.1a	24.1b	31.4b	25.1b	26.9a	58.2b	54.7a	47.6a	53.5a
	80	99.6b	123.9b	163.5b	129.0a	24.3b	32.2ab	26.1ab	27.5a	59.3ab	55.2a	48.1a	54.2a
	95	79.8c	101.9c	129.8c	103.8a	26.9a	34.6a	26.7a	29.4a	61.9a	58.5a	48.2a	56.2a
**Plant spacing treatments (mean value§)**													
	30	149.9a	180.7a	238.9a	189.8a	19.8c	27.6c	22.7c	23.4c	51.0d	43.3d	43.4d	45.9c
	45	109.4b	139.4b	203.1b	150.6ab	20.6c	32.2b	25.1b	26.0bc	53.6d	51.6c	45.7c	50.3c
	60	92.8c	130.0c	166.8c	129.9bc	25.6b	33.3ab	25.2b	28.0b	58.9c	58.9bc	50.3b	56.0b
	75	73.7d	90.6d	124.5d	96.3c	26.5b	33.4ab	26.7b	28.9b	62.3b	60.5ab	47.7c	56.8b
	90	67.8d	80.6e	96.6e	81.7c	32.6a	37.5a	30.2a	33.4a	70.3a	67.0a	52.9a	63.4a

†Data for treatment combination of three row spacing and five plant spacing treatments.

**‡**Row spacing treatments, the same letters in the same column are not significantly different (*P* > 0.05).

**§**Plant spacing treatments, the same letters in the same column are not significantly different (*P* > 0.05).

### 3.2 Racemes per stem

The 3 years average number of racemes per stem of the plant spacing treatments ranged from 23.4 to 33.4 (Table 3). In 2015, the number of racemes per stem plant spacing in 90 cm (32.6) were significantly higher (*P* < 0.05) than that of other treatments. In 2016 and 2017, the number of racemes per stem row spacing of 95 cm were significantly higher (*P* < 0.05) than 65 cm row spacing. Abadouz et al. [20] stated that an increase in the number of racemes with decreasing plant densities (row or plant spacing) can affect alfalfa seed yield. This study showed that the 65 cm row spacing × 60 cm plant spacing was adequate for increasing the number of racemes per stem, pods per stem, and pods per raceme. The decline in the number of racemes per stem and pods per stem with 30 cm plant spacing may have resulted from increased interplant competition for soil nutrients, water, light, and other resources, which eliminated smaller and less vigorous racemes and ultimately increased mortality [17].

### 3.3 Pods per stem

In 2015, the number of pods per stem at 95 cm row spacing was significantly higher (*P* < 0.05) than that of 65 cm row spacing. This represents a 6.4% increase in pods per stem compared to 65 cm (Table 3). In 2016, the quantity of pod production per stem at different row spacing treatments was not obvious, but plant spacing 60, 75, and 90 cm were significantly higher (*P* < 0.05) than the other treatments. The 3 years average number of pods per stem in the plant spacing treatments of 60 and 90 cm were significantly higher (*P* < 0.05) than that of other treatments. Gradual increases in row spacing and plant spacing confronted corresponding decreases in the total quantity of stems per m^2^, but the thinner stands of alfalfa exhibited increased number of racemes and pods per stem. Correlation analysis showed a significant negative correlation between the number of racemes per stem, pods per stem, and stems per m^2^ showing correlation coefficient of −0.798 and −0.822, respectively (*P* < 0.01, Table 4).

**Table 4:** Correlation analysis for seed yield and yield components across row spacing and plant spacing treatments in the three years

Seed yield components	Stems per m^2^	Racemes per stem	Pods per stem	Pods per raceme	Seeds per pod	1,000 seeds weight (g)	Seed yield (kg ha^−1^)
Stems per m^2^	1.000						
Racemes per stem	−0.798**	1.000					
Pods per stem	−0.822**	0.958**	1.000				
Pods per raceme	−0.095	0.419	0.487	1.000			
Seeds per pod	−0.061	0.496	0.496	0.907**	1.000		
1,000 seeds weight	−0.506	0.287	0.408	0.261	0.009	1.000	
Seed yield	−0.223	0.624*	0.656**	0.886**	0.860**	0.200	1.000

*0.05 level significant; **0.01 level significant.

### 3.4 Pods per raceme

The row spacing treatments on the number of pods per raceme showed significant differences during the 3 years of study (Table 5). The number of pods per raceme with row spacing of 65 cm was significantly higher (*P* < 0.05) than row spacing of 95 cm in 2015 and 2017. In 2015, the number of pods per raceme at plant spacing of 90 cm was the highest (10.9). In 2016, the number of pods per raceme with plant spacing of 60 cm was also higher (8.7) significantly (*P* < 0.05) than the plant spacing of 90 cm. This represents a 2.4% increase in pods per raceme compared to 90 cm. These results indicated that the number of pods per raceme were important yield components when the planting density was the primary factor affecting seed yields of alfalfa, as the seed yield composition (pods per raceme) was closely related to pollinator (honeybee and bumblebee) effects [21].

**Table 5:** Average values for pods per raceme, seeds per pod, and 1,000 seeds weight in 2015, 2016, and 2017

Plant spacing (cm)	Row spacing (cm)	Pods per raceme				Seeds per pod				1,000 seeds weight (g)			
		2015	2016	2017	Mean	2015	2016	2017	Mean	2015	2016	2017	Mean value
30	65**†**	8.2	7.4	7.9	7.8	4.0	4.2	5.3	4.5	2.11	2.13	1.96	2.07
	80	8.5	8.5	8.6	8.5	3.9	5.1	5.7	4.9	2.12	2.19	1.94	2.08
	95	6.1	8.2	8.9	7.7	3.3	5.3	6.1	4.9	2.08	2.13	1.82	2.01
45	65	8.8	8.4	10.2	9.1	3.9	5.0	6.3	5.1	2.13	2.21	1.94	2.09
	80	7.7	8.2	9.3	8.4	3.5	5.2	5.6	4.8	2.13	2.19	1.96	2.09
	95	7.6	8.7	9.5	8.6	3.2	5.6	5.7	4.8	2.10	2.22	1.98	2.10
60	65	10.8	9.4	10.4	10.2	4.8	6.4	7.2	6.1	2.14	2.18	1.93	2.08
	80	10.1	9.1	9.6	9.6	4.5	5.5	5.7	5.2	2.12	2.18	1.97	2.09
	95	7.7	7.8	9.3	8.3	3.3	4.4	5.5	4.4	2.08	2.25	2.05	2.13
75	65	9.6	8.9	8.4	9.0	4.2	5.8	6.2	5.4	2.09	2.20	2.01	2.10
	80	9.5	7.9	8.5	8.6	4.2	4.7	5.9	4.9	2.15	2.27	2.02	2.15
	95	7.7	6.8	7.7	7.4	3.3	4.2	5.1	4.2	2.12	2.22	1.98	2.11
90	65	10.2	8.2	10.8	9.7	4.7	5.1	7.6	5.8	2.12	2.24	1.99	2.12
	80	10.9	8.0	8.3	9.1	4.9	4.9	5.5	5.1	2.15	2.18	1.96	2.10
	95	11.5	9.2	7.1	9.3	5.3	6.1	5.3	5.6	2.20	2.23	1.89	2.11
**Row spacing treatments (mean value ‡)**													
	65	9.5a	8.5a	9.5a	9.2a	4.3a	5.3a	6.5a	5.4a	2.11a	2.19a	1.96a	2.09a
	80	9.4a	8.3a	8.8ab	8.8a	4.2a	5.1a	5.7b	5.0a	2.14a	2.20a	1.97a	2.10a
	95	8.1b	8.1a	8.5b	8.2a	3.6b	5.1a	5.6b	4.8a	2.12a	2.21a	1.94a	2.09a
**Plant spacing treatments (mean value§)**													
	30	7.6c	8.1ab	8.4b	8.0b	3.7c	4.8b	5.7a	4.7a	2.10a	2.15b	1.91a	2.05b
	45	8.0c	8.5ab	9.6a	8.7ab	3.5c	5.3a	5.8a	4.9a	2.12a	2.21ab	1.96a	2.10ab
	60	9.5b	8.7a	9.7a	9.3a	4.2b	5.4a	6.1a	5.2a	2.11a	2.20ab	1.98a	2.10a
	75	8.9b	7.9ab	8.2b	8.3ab	3.9bc	4.9b	5.7a	4.8a	2.12a	2.23a	1.99a	2.11a
	90	10.9a	8.5b	8.7ab	9.4a	4.9a	5.3a	6.2a	5.5a	2.15a	2.22a	1.95a	2.11a

†Data for treatment combination of three row spacing and five plant spacing treatments.

**‡**Row spacing treatments, the same letters in the same column are not significantly different (*P* > 0.05).

**§**Plant spacing treatments, the same letters in the same column are not significantly different (*P* > 0.05).

### 3.5 Seeds per pod

Remarkable differences were observed in the number of seeds per pod among plant spacing treatments (Table 5). In 2015, the quantity of seeds per pod at plant spacing of 90 cm was the highest (4.9). The number of seeds per pod of different row spacing was between 4.8 and 5.3. The number of seeds per pod under different plant spacing was between 4.7 and 5.5. The quantity of seeds per pod did not show significant differences with increases in row spacing and plant spacing. This result was consistent with the research findings of Kowithayakorn and Hill [18]. The studies indicated that the number of seeds per pod were not important yield components when the planting density was the primary factor affecting seed yields of alfalfa.

### 3.6 1,000 seeds weight

The 3 years average of 1,000 seeds weight of the plant spacing treatments ranged from 2.05 to 2.11 g (Table 5). The effect of row spacing treatments on 1,000 seeds weight were not significant. Seed weight has been reported to be of minor importance in seed yield [22,23]. This result also supported the findings of Bolanos-Aguilar et al. [24], the alfalfa seed weight per inflorescence may be closely related to genetic factors in alfalfa [25].

### 3.7 Seed yield

**Figure 1:**
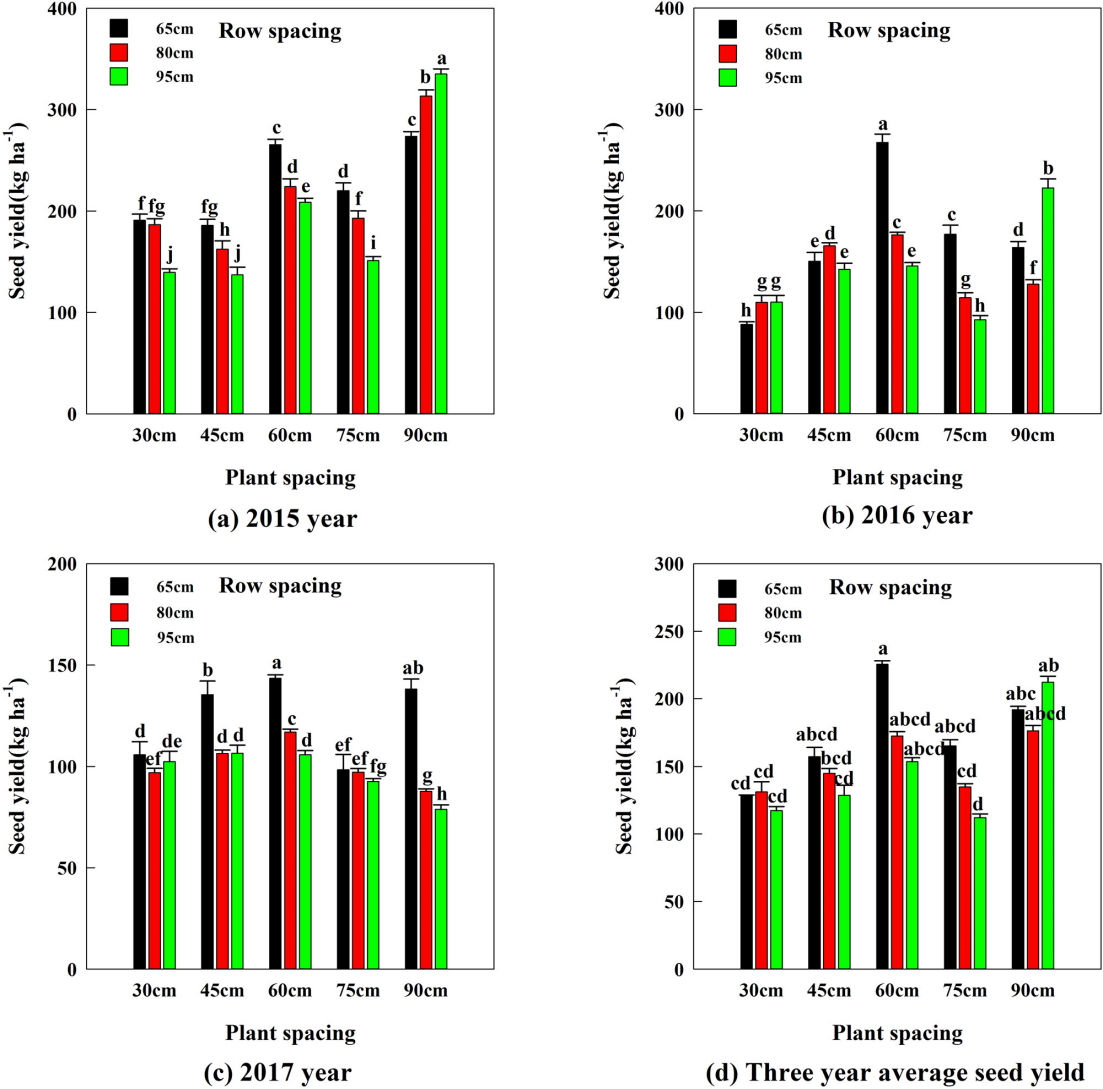
The effects of different row spacing and plant spacing combinations on seed yield. (a) Year 2015, (b) Year 2016, (c) Year 2017, and (d) 3 years average seed yield. Data for row spacing treatments are pooled across plant spacing treatments. Seed yield represented by bars with the same letters in each graph and each treatment is not significant different at 0.05 probability level.

Row spacing and plant spacing treatments on seed yield showed significant differences in 3 years (Figure 1). In 2015, row spacing (95 cm) × plant spacing (90 cm) treatment of seed yield (335.25 kg ha^−1^) was the highest (Figure 1a). In 2016, row spacing (65 cm) × plant spacing (60 cm) treatment of seed yield (267.51 kg ha^−1^) was the highest (Figure 1b). When the spacing in row spacing treatment was 65 cm in 2017, seed yield in plant spacing treatments of 45, 65, and 90 cm was significantly higher (*P* < 0.05) than the other treatments (Figure 1c). The highest 3 years average seed yield was obtained with row spacing of 65 cm × plant spacing of 60 cm, which was 225.49 kg ha^−1^ (Figure 1d). Thinning done to reduce plant density (row or plant spacing) has long been known to improve the seed yield of alfalfa [9]. Previously, Askarian et al. [15] found that alfalfa seed yield obtained with 15 cm row spacing was significantly less than those of the 30, 45, and 60 cm. Zhang et al. [17] also reported that intermediate planting density treatments (80 cm row spacing and 30 cm plant spacing) obtained the highest seed yields over 4 years. These results support the view of Duro et al. [26] that alfalfa should be grown in row spacing 60 cm, which was most productive for increasing seed yield. On the one hand, plants in intermediate density stands have ample room and resources to increase seed yield by increasing more branches, racemes, and pods per stem [16,27]. The probability of flowering generally increases with the distance of the plant, thus indicating an increasing resource availability of plants [26]. On the other hand, high plant density treatments could result in a negative effect on alfalfa seed yields [18], such as row spacing (65 cm) × plant spacing (30 cm) produced greater interplant competition, thereby easily causing the plant lodging and affecting the insect pollination. Kowithayakorn and Hill [18] reported that alfalfa seed production relies upon seed yield per plant rather than the number of plants per unit area, and low plant density (wide row or plant spacing) promotes more branches, racemes per stem, higher percentage seed pod, and higher alfalfa seed yields. However, there was a point of diminishing yields whereby higher yields per plant cannot compensate for the lower plant density. The lower plant density not only causes waste of land room, light, water, and other resources, but also fails to obtain optimal seed yield in later years [28]. In conclusion, the highest alfalfa seed yield depends on the best balance between row spacing and plant spacing, rather than either of these two factors individually.

The actual seed yield accounts for only a proportion of the potential seed yield on the standing alfalfa, which are due for harvest [6]. There were two probable reasons. First, the loss of seed yields mainly resulted from the processes of cutting, threshing, and cleaning alfalfa, as those were done using different hand tools. Second, alfalfa is a cross-pollinated plant whose pollination is carried out with the help of insects (honeybee), wind, and other external factors [8]. So, it could be concluded that the seed yield in alfalfa is usually affected because of climatic conditions, low temperature, and high humidity associated with excess precipitation during flowering, which limited insect pollination and increased physiological losses of pollinated flowers [24,25].

## 4 Conclusion

This study concluded a remarkable positive correlation between the number of racemes per stem, pods per stem, pods per raceme, seeds per pod, and seed yield. The highest alfalfa seed yields were obtained with 65 cm row spacing and 60 cm plant spacing over a 3 years period of study. Therefore, this study suggested that alfalfa should be cultivated by using 65 cm row spacing and 60 cm plant spacing to maximize seed yields in the western Heilongjiang areas, China.
